# Selectivity of timing: A meta-analysis of temporal processing in neuroimaging studies using activation likelihood estimation and reverse inference

**DOI:** 10.3389/fnhum.2022.1000995

**Published:** 2023-01-05

**Authors:** Chloe Mondok, Martin Wiener

**Affiliations:** Department of Psychology, George Mason University, Fairfax, VA, United States

**Keywords:** temporal processing, meta-analysis, ALE, reverse inference, SMA

## Abstract

Over the last few decades, many researchers have investigated time perception and how it is processed in the brain. Past studies have identified cortical and subcortical regions that play an important role in implicit and/or explicit timing tasks. In regard to timing, different regions appear to have roles of varying importance depending on the duration (sub-second vs. supra-second), type of task (such as involving motor responses or passively observing stimuli), and modality (such as auditory, visual, and sensorimotor) resulting in the literature reporting divergent results that are contingent on the specifics of the task. This meta-analysis aims at identifying regions that show activation only for explicit timing tasks through reverse inference. As such, two datasets (the first including studies that involved explicit timing tasks while the second did not) were compared using the activation likelihood estimation (ALE) algorithm. Reverse inference was implemented through Bayes factor modeling, which allowed for the comparison of the activated regions between the two ALE-maps. Results showed a constellation of regions that exhibited selective activation likelihood in explicit timing tasks with the largest posterior probability of activation resulting in the left supplementary motor area (SMA) and the bilateral insula. Some areas that have been dubbed critical for time perception in past studies (i.e., the cerebellum) did not exhibit prevalent activation after analyses.

## Introduction

For a number of years, it has been debated whether the brain consists of numerous “clocks” in which designated areas are responsible for maintaining and perceiving time intervals, or if time was a shared ability among neural networks (Merchant et al., 2013). Notably, a harmony of these two theories has become the most acceptable theory in which temporal processing consists of a distributed network that includes both cortical and subcortical structures, with regions that are specialized for timing under specific circumstances (Nachev et al., 2008; Coull et al., 2015; Cona and Semenza, 2017; Mioni et al., 2020). Further, it is suggested that the brain utilizes a core system of structures and may recruit secondary areas that are dependent on the present condition and are altered based on time-dependent changes (Grondin, 2010; Merchant et al., 2013; Merchant and Yarrow, 2016). This core system is thought to be comprized of areas that are part of the motor system including the cerebellum, SMA, premotor cortex, basal ganglia (Teki et al., 2012; Allman et al., 2014) and, in particular, the corticothalamic-basal ganglia timing circuit (CTBGc; Merchant et al., 2013; Merchant and Yarrow, 2016). It is through this circuit that the thalamus sends information between the cortex and basal ganglia, allowing for dynamic temporal prediction, especially in regard to perceptual integration (Schwartze and Kotz, 2013). In addition, areas that are a part of higher-order cognition including networks responsible for attention and working memory (Coull et al., 2008; Allman et al., 2014; Radua et al., 2014) such as the prefrontal cortex (Teki et al., 2012; Allman et al., 2014) are also involved. As noted in previous literature, this core network can be further divided into striatal and olivo cerebellar networks that work in tangent (Teki et al., 2012; Allman et al., 2014), placing a large focus on the role of the basal ganglia and the cerebellum in temporal perception.

Investigations into how time is processed in the brain is accomplished in two ways: explicit and implicit timing tasks. For explicit timing tasks, the goal of the participant is to estimate a specific amount of time (dependent on the parameters of the task) in relation to the interstimulus interval (ISI) through either motor timing or perceptual timing tasks (Coull and Nobre, 2008). Contrarily, implicit timing tasks refer to tasks whose goal is not centered on timing, though covert timing is a component and can be comprized of emergent timing through motor outputs as well as temporal expectations through perceptual input (Coull and Nobre, 2008). For the purposes of this meta-analysis, we will be utilizing the definition of explicit timing when referring to time perception, especially in regard to the experiments included in the “IS TIME” dataset in the Methodology which involved sub-second and supra-second intervals as well as motor and perceptual tasks.

Numerous factors may play a role in which neural networks are activated during explicit timing tasks, such as task type and duration. For example, motor timing tasks (i.e., production and reproduction) compared to perceptual timing tasks (i.e., temporal discrimination) involve different overlapping neural networks; likewise, duration, in regard to sub-second and supra-second time intervals, have also been shown to engage different regions leading to divergent findings depending on experimental methodology (Wiener et al., 2010).

It is noteworthy to mention that additional factors may distort the magnitude of activation for time perception, an example being attention, in which attentional deficits (Mioni et al., 2013) have shown less activation as well as altered perceptions of duration length (Grondin, 2010). In fact, other cognitive functions such as working memory and sequencing are utilized by the core timing network, resulting in changes in activated regions dependent on experimental tasks, leading to discrepancies in the literature (Livesey et al., 2007; Radua et al., 2014).

Despite the increase in recent publications pertaining to temporal perception, it is still unclear how the brain processes time. One obstacle may be the conditional elements of experiments which vary among researchers, making it harder to generalize results. Given that regions involved in time perception are also involved in other domains, different activation patterns may arise between studies. That being said, past reviews have found similarities among findings, allowing for a more comprehensive look at temporal processing.

Given that the timing neural network greatly overlaps with the motor network, investigating the differences in activation patterns across motor and perceptual timing tasks have become more common. Wiener et al. (2010) ran a quantitative meta-analysis using the Activation Likelihood Estimation (ALE) algorithm comparing type of task (motor vs. perceptual) and stimulus duration (sub-second vs. supra-second). The authors reported that there were distinct sub-networks of brain regions depending on the task context, across frontal and parietal cortices, as well as sub-cortical regions within the basal ganglia and cerebellum; for example greater sub-cortical involvement was observed for sub-second intervals. Further, significant activation likelihood in the bilateral SMA and right inferior frontal gyrus were observed regardless of task (perceptual vs. motor tasks) and duration (sub- vs. supra-second). Similarly, Nani et al. (2019) ran an ALE meta-analysis using updated methods that compared activation patterns for task (motor vs. non-motor) and duration (sub-second vs. supra-second). These authors found high overlap for both ranges of duration in the superior frontal gyri, medial frontal gyri, middle frontal gyri, and inferior frontal gyri, SMA, and claustrum (Nani et al., 2019). Though the neural networks for the different tasks and durations overlapped greatly, they found that subcortical regions seemed to be more involved in motor tasks and sub-second durations while cortical regions showed more involvement with the perceptual tasks and supra-second durations, corroborating earlier findings (Nani et al., 2019).

Similarly, neural networks for timing have been shown to be influenced by other networks outside of the system, such as those involved in executive functioning. Radua et al. (2014) ran a neuroimaging meta-analysis using signed differential mapping (SDM), comparing activation likelihood in time perception and executive function and found that both domains overlapped in the fronto-parietal-insula and putamen, suggesting that temporal processing involves cognitive functions such as attention and working memory, or at least that these functions could not be reliably separated from one another. Yet, the researchers found areas that were involved in time perception but not executive functioning, suggesting that time perception has its own neural network which may overlap with other domains (Radua et al., 2014). They also found that activity for certain regions involved in time perception was modulated depending on task load such that tasks that were deemed more difficult evoked more activation in the SMA, insula/operculum, dorsolateral prefrontal cortex, thalamus, and striatum (Radua et al., 2014). Similarly, Livesey et al. (2007) also observed that the left inferior parietal lobule, pre-SMA, and part of the dorsal prefrontal cortex only had significant activation in larger task loads that was absent in less difficult tasks. Both of these studies show how the differences in experimental tasks can modulate activation for time perception.

Though the current literature demonstrates a myriad of experiments exploring explicit and implicit timing, it is important to consider methodology, especially in regard to discrepancies between studies. For example, Mioni et al. (2020) demonstrated that experimental modality plays an important role when examining the neural networks at play for time perception. Many of the aforementioned studies utilized functional magnetic resonance imaging (fMRI) to examine neural activity while Mioni et al. (2020) ran their analysis on temporal studies that used transcranial magnetic stimulation (TMS); they found that some prominent areas that were agreed upon from other studies to be integral to temporal processing, were not seen in their review. One area to note is the SMA, which did not show changes depending on motor or perceptual tasks in the TMS studies used, stating that its connections with subcortical regions (which are not stimulated during TMS) may be the cause (Mioni et al., 2020). Yet, the other possibility is that the SMA is not critical for timing, and so its prominent observation across meta-analyses may in fact be epiphenomenal. Indeed, the SMA and right prefrontal cortex, both areas observed to be active across all timing contexts, are also commonly observed across the larger corpus of neuroimaging studies in general (Behrens et al., 2013). As such, their involvement in timing studies is called into question.

Thus, how to address the question of the necessity for timing? While useful, all of the previous meta-analyses described have relied on forward inference. That is, activation-likelihood was tested for a particular function by aggregating studies that studied that function. In this case, any regions that survive significance testing are those that are most likely to be active for that particular function. In the case of timing, these meta-analyses can only say if a region will be active given that a subject performs a timing task; for example, the SMA activation likelihood can be construed as p(SMA| timing). Yet, activation likelihood says nothing regarding the specificity of observed regions for a particular function. That is, if activity were observed in a given region first, what is the probability that subjects were engaged in that function? Using the above example, for the SMA this would be inferred as p(timing| SMA); that is, if activity is observed in the SMA, what is the probability the subject was timing? This line of reasoning forms a so-called reverse inference (Poldrack, 2006), a common interpretive pitfall in neuroimaging studies. However, for meta-analyses, the problem can be formulated using Bayes Theorem to calculate the posterior probability of activation. This method was recently developed as an addition to the ALE methodology (Costa et al., 2021), and can be employed by calculating the posterior probability across two ALE maps: one for the function in question (in this case, time perception), and one not for that function (in this case, studies not on time perception); this use of posterior probability distinguishes this meta-analysis from other timing meta-analyses. Thus, the present study applied this methodology to neuroimaging studies of time perception to investigate the specificity of timing regions using posterior probability.

## Methodology

### Literature search

The literature search consisted of searching the BrainMap database.^1^ First, Sleuth 3.0.4^2^ software provided through BrainMap was used to discover the relevant articles. For this meta-analysis, two datasets were collected: the first being articles that investigated explicit timing (“IS TIME”) with the second being articles that did not investigate explicit timing (“IS NOT TIME”). The “IS NOT TIME” dataset was created through Sleuth *via* the following search algorithm:

IS NOT TIME: [*Experiments Context IS Normal Mapping] AND [Experiments Activation IS Activation Only] AND [Subjects Diagnosis IS Normals] AND [Experiments Imaging Modality IS fMRI] AND [Experiments Imaging Modality IS PET] AND [Experiments Behavioral Domain IS NOT Cognition/Temporal]*.

In this way, BrainMap was utilized for its ability to search for an array of articles that did not include explicit timing tasks (as denoted by the “IS NOT” in the Sleuth pathway). BrainMap provides a unique opportunity to collect a vast number of articles outside of our target function (time perception) which allowed for a comparison to the “IS TIME” dataset.

The “IS TIME” dataset was provided from Cona et al. (2021), a recent study investigating time perception and comparing it with spatial processing; the “IS TIME” meta-analysis included 114 experiments, 1,262 foci, and 1,703 participants. These studies involved explicit timing tasks which were collected according to the PRISMA guidelines. The “IS NOT TIME” dataset, which was acquired through Sleuth, consisted of 9,953 experiments, 84,143 foci, and 155,041 participants and did not involve explicit timing tasks. Both datasets were cross-referenced to remove duplicate studies that were included in both groups.

These datasets were then analyzed through GingerALE 3.0.2 from BrainMap^3^ using the Talairach coordinate system. All information in MNI (Montreal Neurological Institute) coordinates were automatically converted to Talairach through GingerALE, which was used to create unthresholded modeled activation maps for both datasets.

### Activation likelihood estimation

The Activation Likelihood Estimation (ALE) algorithm is commonly used for quantitative meta-analyses of neuroimaging results. ALE works by modeling activation foci described in each study into three-dimensional Gaussian probability distributions per voxel (Turkeltaub et al., 2011). The overlap of these distributions results in an ALE score which is compared to numerous null distributions that are randomly generated in order to assess significance of the activation foci *via* a permutation test (Wiener et al., 2010). As such, the resultant ALE map provides information pertaining to the probability of overlap of the statistically significant activation foci across studies (Nani et al., 2019; Teghil et al., 2019).

### Reverse inference

**FIGURE 1:**
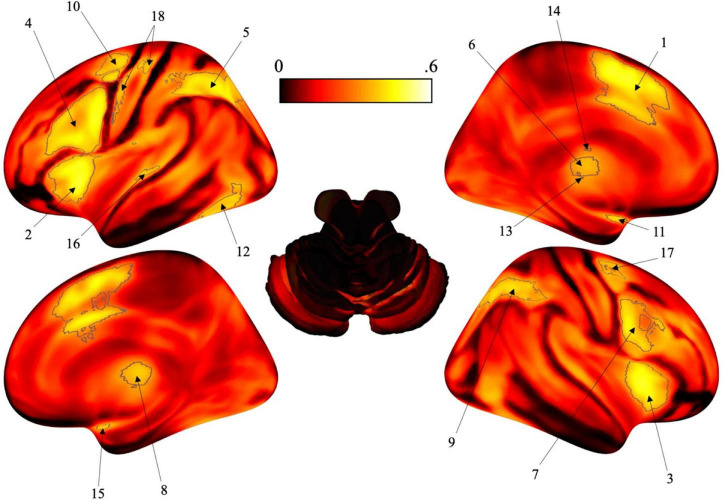
Surface brain renderings of the activation likelihood estimation (ALE) maps. Gray contours represent the areas that have a posterior probability of 0.4 or greater. To note, these posterior probabilities reflect the probability of each individual voxel, not the probability of a cluster (or region) being activated. (1) Left posterior-medial frontal gyrus (SMA). (2) Left insula lobe. (3) Right insula lobe. (4) Left inferior frontal gyrus (pars opercularis). (5) Left angular gyrus. (6) Left thalamus. (7) Right inferior frontal gyrus (pars opercularis). (8) Right thalamus. (9) Right angular gyrus. (10) Left pre-central gyrus. (11) Left amygdala. (12) Left fusiform gyrus. (13) Left putamen. (14) Left caudate nucleus. (15) Right amygdala. (16) Left middle temporal gyrus. (17) Right pre-central gyrus. (18) Left post-central gyrus.

The ALE “IS TIME” and “IS NOT TIME” maps were processed using Mango 4.1 (Multi-image Analysis GUI^4^) software. The Bayes fACtor mOdeliNg (BACON) plug-in for Mango is a computational tool used for reverse inference for neuroimaging data, detailed by Costa et al. (2021). The BACON plug-in was utilized to incorporate the non-timing activation likelihood (“IS NOT TIME” dataset) from the activation likelihood for timing (“IS TIME” dataset) to calculate a posterior probability. As such, there are no overlapping functions in the final map (Figure 1).

The BACON plug-in is rooted in Bayesian statistics since it is heavily based on the Bayes factor. Bayes’ theorem has been used in neuroimaging studies to determine the posterior probability of activation (D) for a voxel in a cognitive process (Cauda et al., 2020; Costa et al., 2021). In this way, reverse inference can be used to infer activation patterns for timing using alterations of Bayes’ theorem. Following equations provided by Costa et al. (2021), Bayes’ theorem can be written expressing two hypotheses: H_0_ (i.e., the presence of a function such as time perception) and H_1_ (i.e., the absence of the function):


$$P\,\left(H_{0}|D\right)=\frac{P\,\left(D|H_{0}\right)}{P\,\left(D\right)}\,P\,\left(H_{0}\right)$$

and


$$P\,\left(H_{1}|D\right)=\frac{P\,\left(D|H_{1}\right)}{P\,\left(D\right)}\,P\,\left(H_{1}\right)$$

Using these equations, P(H_0_| D) and P(H_1_| D) are the posterior probability while P(D) is the prior probability (Cauda et al., 2020). If no foreknowledge for P(H_0_) and P(H_1_) is known, then P(H_0_) and P(H_1_) are considered identical and can be written in the form of Bayes’ factor:


$$B\,F_{01}=\frac{P\,\left(H_{0}|D\right)}{P\,\left(H_{1}|D\right)}$$

This equation allows one to compare the hypotheses such that if BF > 1, then H_0_ is supported while the opposite is true in that if BF < 1, then H_1_ is supported. Further, since both posterior probabilities summate to 1, the equation can be written in its final form as noted by Costa et al. (2021):


$$P\,\left(H_{0}|D\right)=\frac{B\,F_{01}}{B\,F_{01}+1}$$

As such, the BACON plug-in includes this final equation to compute the posterior probability for activation in a specific cognitive process such as time perception (Costa et al., 2021). Through Mango, it compares ALE maps (“IS” and “IS NOT”) to calculate the independent activation of the foci allowing for reverse inference.

## Results

After analysis, selective activation for time perception was found bilaterally in the insula, pars opercularis, angular gyrus, thalamus, pre-central gyrus, and amygdala. Some activation was localized to the left hemisphere including the posterior-medial frontal gyrus, fusiform gyrus, putamen, caudate nucleus, medial temporal gyrus and post-central gyrus (see Figure 1 and Table 1).

**TABLE 1:** Brain regions with selective activation for timing tasks in neuroimaging studies using the activation likelihood estimation (ALE) algorithm.

Location	Volume (mm^3^)	Posterior probability	MNI coordinates		
			x	y	z
Left posterior-medial frontal gyrus (SMA)	1,437	0.483	−2	6	50
Left insula lobe	2,018	0.481	−32	18	6
Right insula lobe	581	0.478	32	20	4
Left inferior frontal gyrus (pars opercularis)	2,018	0.473	−44	4	30
Left angular gyrus	627	0.453	−30	−56	42
Left thalamus	187	0.452	−12	−18	8
Right inferior frontal gyrus (pars opercularis)	299	0.449	44	6	30
Right thalamus	115	0.434	8	−16	8
Right angular gyrus	250	0.427	28	−58	44
Left pre-central gyrus	87	0.424	−28	−8	52
Left amygdala	50	0.422	−22	−4	−12
Left fusiform gyrus	116	0.415	−40	−58	−14
Left putamen	69	0.411	−14	6	2
Left caudate nucleus	59	0.410	12	6	4
Right amygdala	7	0.407	22	−4	−12
Left middle temporal gyrus	11	0.404	−56	−20	6
Right pre-central gyrus	9	0.403	28	−6	52
Left post-central gyrus	6	0.403	−36	−24	52

Shows all local maxima separated by more than 20 mm. Regions were automatically labeled using the Anatomy Toolbox atlas. x, y, and z = Montreal Neurological Institute (MNI) coordinates in the left-right, anterior-posterior, and inferior-superior dimensions, respectively.

The area that exhibited the highest posterior probability [p(timing| activation)] for timing tasks was the left posterior-medial frontal gyrus (SMA at 0.483) while the lowest was the left post-central gyrus (0.403). Following the left SMA, the areas with the highest posterior probability were mainly located in the left hemisphere and included the left insula (0.481), right insula (0.478), left pars opercularis (0.473), left angular gyrus (0.453), and left thalamus (0.452). The remaining areas that exhibited selective activation for timing tasks did not exceed a posterior probability of 0.45 and are located in Table 1.

## Discussion

After running the ALE algorithm, 18 regions were identified as having the relatively highest posterior probability during timing tasks using reverse inference (Table 1). Though the 0.4 threshold was chosen arbitrarily to generate clearly delineated clusters, the posterior probability values of each region allow for the comparison of the *relative difference in probability* from one region to another. Indeed, we suggest that the utility of our analysis is to reveal differences between the probability of activation for different brain regions during timing tasks. Thus, for anyone conducting a neuroimaging study of time perception, they may consider the probability that any one brain region will be observed in their study. Numerous areas that are known to be a part of the neural network for processing time were supported in the data including parts of the motor circuit such as the SMA and pre-central gyrus, subcortical structures such as the thalamus, as well as various cortical areas across the frontal, parietal, temporal, and occipital lobes. From our analysis, the highest posterior probability was found in the left SMA at 0.483 when controlling for activity in non-timing tasks.

The SMA is located in the dorsomedial frontal cortex and is comprized of the pre-SMA and SMA-proper (Nachev et al., 2008). Most notably, the SMA is a key component of the neural network for timing but is also involved in other domains outside of time perception including motor planning and action sequencing since it connects to areas including the basal ganglia, primary motor cortex, and prefrontal cortex (Nachev et al., 2008; Cona and Semenza, 2017). Since the SMA is involved in both motor and non-motor tasks, Cona and Semenza (2017) proposed the domain-general hypothesis in which the SMA is involved in one operation across different domains, such as time, space, language, memory, etc., all of which the SMA is known to play a role in. Wiener et al. (2010) provides evidence to support the domain-general hypothesis in that the SMA exhibited significant activation for timing tasks regardless of stimuli duration or condition, allowing the SMA to have a versatile role in temporal processing. In this way, the SMA may be involved in numerous cognitive domains in an energy efficient manner through sequence processing as the common operation for each of these domains (Tanji and Shima, 1994; Gerloff et al., 1997; Menon et al., 2000). Though sequence processing is important for multiple domains, it is imperative for an internal representation of time. As shown by Tanji and Shima (1994), the SMA showed increased activity for certain sequences of movements in trained Japanese Macaques in the absence of external guidance, which supports the SMA’s role in sequencing in motor planning and execution. In addition, Menon et al. (2000) showed changes in activation specifically in the left pre-SMA depending on the number of operands in a numerical problem such that mathematical problems involving three operands elicited greater activity than those involving two, supporting the SMA’s role in sequencing. This is further supported by the SMA’s involvement in maintaining rhythm. It does so by acting as an internal metronome for processing rhythms (Cannon and Patel, 2021), in which the SMA matches rhythms by entraining firing rates to the perceived beat, replicating the beat internally (Cadena-Valencia et al., 2018; Cannon and Patel, 2021). As such, the SMA has shown higher activation levels for rhythms that maintain the beat compared to irregular rhythms with non-beats (Grahn and Rowe, 2012).

Though the SMA had the highest posterior probability of being invoked during timing tasks (0.483 for the left SMA) in this analysis, it does not show as much as activity as others have previous published. That being said, Wiener et al. (2010) and Radua et al. (2014) both found the SMA (BA 6) to have the highest level of activation voxel-wise for time perception. It is important to note recent studies have found evidence of a gradient in the SMA in regard to spatial and temporal processing (Cona et al., 2021). Cona et al. (2021) used ALE to find a spatial-temporal gradient in the SMA in which parts of this area processed both time and space. Since this analysis effectively removed any activity outside of that involving timing, this may help explain why the percentage of activity is somewhat smaller in this analysis in regard to other publications, though additional factors may also be at play.

Another region that had relatively high posterior probability was the bilateral insula. Similar to the SMA, the insula has recently been considered to function as an accumulator for timing as shown in a study by Wittmann et al. (2010), in which participants listened to one of three supra-second tones (the encoding phase) and then reproduced that duration through a button press (reproduction phase). They found that the posterior insular cortex, as well as other regions such as the SMA, were activated for the encoding phase while the anterior insula (and other areas of the anterior cortex) was activated for the reproduction phase, suggesting a neural network for processing supra-second intervals in which the posterior regions of these areas encode durations while the anterior regions maintain those durations (Wittmann et al., 2010). In addition, a review by Vicario et al. (2020) noted that there is a relationship between altered levels of insular activation in clinical disorders such that participants with disorders that displayed time underestimation (such as those with Parkinson’s disease, autism, and attention deficit hyperactivity disorder) also had reduced levels of activity in the insula while those that displayed time overestimation (i.e., schizophrenia, depression, and anxiety) had increased levels of insular activity. Further, Vicario et al. (2020) also noted that insula activity was found specifically during supra-second timing tasks, reiterating the insula’s role in timing across numerous seconds.

Overall, a number of regions in the frontal and parietal lobes have shown to play a role in temporal processing. Fedorenko et al. (2013) introduced the multiple-demand system as a way to explain how numerous regions in the frontal and parietal areas appear to contribute to a variety of cognitive functions such as temporal and spatial processing, attention, performance monitoring, and so forth. Further, additional frontal regions, such as the inferior and middle frontal gyri are also recruited for temporal processing, most likely because of high-level cognition such attention and working memory (Nani et al., 2019; Cona et al., 2021). Further, Wiener et al. (2010) also found that the right inferior frontal gyrus (as well as the SMA) showed significant activation regardless of task and duration, supporting the idea that frontal regions also play a role in motor timing tasks, not just perceptual timing tasks. As an extension of the multiple-demand network, Camilleri et al. (2018) recently conducted a larger, hierarchical meta-analysis which provided distinctions in activation likelihood across distinct regions. Here, the SMA is likely to be invoked as an organizational hub for task demands; that is, depending on the task domain (i.e., language, working memory, timing), the SMA recruits other regions to perform that task. Indeed, other meta-analyses of time perception studies have demonstrated that activation likelihood in the SMA can be fractionated on the sensorimotor aspects of the task, such that more anterior regions of the SMA complex are likely to be recruited for perceptual timing tasks (where no motor response is required), whereas posterior regions are preferentially recruited for motor timing tasks (where a motor response demarcates the timed interval) (Wiener et al., 2011; Schwartze et al., 2012). In contrast, other regions of the network, including the IFG and inferior parietal cortex serve as “workers”, such that these regions abstract the rules and decisions of the tasks for higher-order cognition.

Notably, there are a number of regions that are believed to be a core component of the timing neural network that did not have a large relative difference in activation in this analysis. This may be due to a number of reasons including the fact that this analysis used reverse inference to find activation selectivity for timing tasks while brain regions that are known to be involved with timing may have shown equal or higher activation in non-timing tasks compared to timing tasks. An example is the cerebellum, which is known to play a considerable role in temporal processing (Teki et al., 2012; Allman et al., 2014; Coull et al., 2015; Paton and Buonomano, 2018; Mioni et al., 2020), though it did not have relatively high probability in this analysis.

The cerebellum is primarily known for its involvement in motor functions such as coordinating movements. O’Reilly et al. (2008) provided evidence that the anterior portion of the cerebellum is the primary area for motor-related functions while the posterior cerebellum is more so involved in non-motor functions. When exploring temporal-spatial models in the cerebellum, O’Reilly et al. (2008) found that part of the posterior cerebellum was involved in judging timing. One of the ways in which the cerebellum is known to be involved with temporal processing is through music, specifically by maintaining the beat. The cerebellum is able to perform beat maintenance by projecting to frontal regions using forward prediction models in which it is able to anticipate the beat and respond to errors, similar to the SMA (Cadena-Valencia et al., 2018; Cannon and Patel, 2021). To support this, Fujioka and Ross (2017) found increased event-related desynchronizations in beta oscillations for piano players compared to those without training for shorter beat intervals (400-ms) compared to longer beat intervals (1,200-ms) not only in the cerebellum, but also in other regions involved in the motor network including sensorimotor and premotor cortices. This also supports the cerebellum’s preference in sub-second durations compared to supra-second durations (Wiener et al., 2010; Fujioka and Ross, 2017; Mioni et al., 2020). On the other hand, Radua et al. (2014) did not find activation for the cerebellum in regard to explicit time perception but only for higher cognitive functions, suggesting that these higher-level functions overlap with those required for processing time. That being said, the cerebellum is greatly involved in the motor network, which may at least partially explain its lack of activity in this analysis.

Another region of the brain that has been well documented as a contributor to temporal processing is the basal ganglia, a collection of subcortical nuclei primarily known for being a main component of the motor network and important for sensory timing and the timing of motor movements (Coslett et al., 2010; Paton and Buonomano, 2018). Structurally, damage to the basal ganglia causes changes in timing in numerous disorders such as Parkinson’s disease, Huntington’s disease, attention-deficit disorder, and more (Paton and Buonomano, 2018); yet, damage to the basal ganglia does not always equate to a failure in temporal processing (Coslett et al., 2010). The basal ganglia have been attributed with creating an internal sense of time through the use of predictions (Grahn and Rowe, 2012; Paton and Buonomano, 2018). An example of which the basal ganglia has been shown to achieve this has been shown through its role in maintaining a metric hierarchy since it shows higher activation in regard to duple meter compared to triple meter as well as irregular rhythms that break the meter (Grahn and Rowe, 2012). It is not surprising that the basal ganglia is able to make temporal predictions since timing is imperative to movement sequences and is subsequently reinforced (Paton and Buonomano, 2018). It is believed that the basal ganglia shares its prediction models closely with the cerebellum given their direct connection through the striatum (O’Reilly et al., 2008). Further, publications have claimed that the basal ganglia is imperative to timing for both motor and sensory modalities (O’Reilly et al., 2008; Paton and Buonomano, 2018) while others provide a more supportive role for the basal ganglia in regard to timing (Coslett et al., 2010) so its true contribution is still rather elusive.

### Limitations

Our analysis utilized reverse inference through the ALE algorithm to essentially subtract selective activity for temporal tasks from non-temporal tasks. The analysis was accomplished *via* the BACON plug-in which accounts for activation-likelihood against the target activation-likelihood. As such, the analysis is not excluding undifferentiated functions per say, but rather incorporating both likelihoods into an estimate of posterior probability. Nevertheless, regions that may be involved in non-timing processing still had a relative difference in activation in timing tasks since these regions exhibited more activation likelihood for timing than non-timing tasks across the studies included. The BACON analysis is sensitive to different levels of activation likelihood, which is why some multi-modal areas did not have a relatively high posterior probability (i.e., cerebellum) while some did (i.e., SMA). Thus, one of the cons of using this methodology is the fact that activity in multi-modal areas is partially or, in some cases, fully lost after analysis, as demonstrated by the lack of prevalent data in the cerebellum, which is a large, heterogeneous structure, which may make it more difficult to find convergence in these areas across studies considering the enormity of its non-timing activity.

In line with this, a limitation of this analysis can be attributed to dataset size. The “IS TIME” dataset (which included 114 experiments involving explicit timing tasks) was quite smaller than the “IS NOT TIME” dataset (which included 9,953 experiments involving non-timing tasks). As such, the enormity of the non-timing activity in the “IS NOT TIME” dataset may have biased the activation levels in favor of the non-timing related activity, overshadowing the timing related activity. Because of this size difference, the raw ALE values for the “IS NOT TIME” dataset were an order of magnitude larger than those for the “IS TIME” dataset. Further, this may lead to a decrease in the size of the posterior probabilities, as the likelihood of any one voxel will be overall higher for “IS NOT TIME” dataset compared to the “IS TIME” dataset. As a result, the posterior probability of voxel activation for timing seemed low (*p* = 0.4) compared to other publications, which may be attributed to the difference in these ALE values. That being said, this “low” posterior probability for timing may suggest that timing functions are not a special property of any specific part of the brain. Overall, though the “IS TIME” dataset contained much fewer studies than the “IS NOT TIME” dataset, we are still seeing a pattern of relative activity for timing tasks across the brain.

Other possible limitations may lie in the studies themselves with the type of task involved. As suggested by Wiener et al. (2010), the inclusion of sub-second and supra-second timing intervals in the studies included in the “IS TIME” dataset may show different patterns of activity. This may be the case considering Merchant and Yarrow (2016) theory in which the timing neural network is divided into a core network that is constantly activated and a supplementary network that is activated in the presence of certain tasks (in regard to duration, stimulus modality, etc.). In this way, differential aspects of each experiment (i.e., hypothesis, task conditions, stimuli, etc.) naturally cause limitations when comparing studies. Lastly, individual factors that are outside of the authors’ control may also affect the activation levels. For example, it has been noted that attention can greatly affect activity in the neural network for timing, leading to less activation with lower levels of attention across studies (Coull et al., 2008; Allman et al., 2014; Paton and Buonomano, 2018).

## Conclusion

This review investigated activation for time perception in neuroimaging studies using reverse inference through ALE. The analysis showed 18 regions that had a larger relative difference in activation for timing tasks compared to non-timing tasks, with the highest level of activation being in the left SMA. Though the data supported the involvement of parts of the motor network in time perception, some key areas that are known to be a core region essential for time perception were absent. These results demonstrate how regions previously shown to be involved in timing may be more involved in non-timing tasks, suggesting the presence of a domain-general system (Wiener et al., 2010; Cona and Semenza, 2017). As such, hypotheses like the multiple-demand system (Fedorenko et al., 2013) may play a larger role than previously thought.

## Data availability statement

Publicly available datasets were analyzed in this study. This data can be found here: www.brainmap.org.

## Author contributions

MW and CM conceived of the study. CM collected the data, ran the analyses, and wrote the manuscript with guidance from MW. Both authors contributed to the article and approved the submitted version.

## Footnotes

1
www.brainmap.org

2
http://brainmap.org/sleuth/

3
http://brainmap.org/ale/

4
http://ric.uthscsa.edu/mango/
